# Transorbital neuroendoscopy-assisted resection of a giant optic pathway glioma in a neonate

**DOI:** 10.1007/s00381-023-05986-1

**Published:** 2023-05-16

**Authors:** Rodney Maseko, Maanda Mabogo, Zarina Lockhat, Priscilla Makunyane, Samia Ahmad, Meshack Bida, Llewellyn Padayachy

**Affiliations:** 1 Department of Radiology, Faculty of Health Sciences, University of Pretoria, Steve Biko Academic Hospital, Pretoria, South Africa; 2 Department of Ophthalmology, Faculty of Health Sciences, University of Pretoria, Steve Biko Academic Hospital, Pretoria, South Africa; 3 Pediatric Neurosurgery Unit, Department of Neurosurgery, Faculty of Health Sciences, University of Pretoria, Steve Biko Academic Hospital, Pretoria, South Africa; 4 Department of Anatomical Pathology, Faculty of Health Sciences, University of Pretoria, Steve Biko Academic Hospital, Pretoria, South Africa

**Keywords:** Optic pathway glioma, Transorbital neuroendoscopic surgery, Giant orbital tumor, Minimally invasive surgery

## Abstract

Congenital giant orbital tumors in infancy are relatively rare, especially when the tumors are associated with significant intracranial extension. We describe the use of a transorbital neuroendoscopy-assisted resection of such a lesion. While this approach is increasingly gaining popularity for certain anterior and middle skullbase lesions in adults, this report represents the youngest patient reported on where this minimally invasive approach has been successfully used to resect the intracranial tumor. This surgical approach obviated the need for a separate craniotomy, with the additional benefit of minimizing blood loss.

## Introduction

Optic pathway glioma (OPG) is a benign low-grade neoplasm of the optic pathway and is the commonest optic nerve tumour comprising 4% of orbital tumours in children [1]. The neoplasm is largely categorized as either arising from the optic nerve or the surrounding sheath and can involve the optic chiasm, tract, radiations and hypothalamus. OPG is classified by the current 2021 World Health Organization (WHO) [2] Classification of Central Nervous System Tumours under the category of circumscribed astrocytic gliomas, grade 1 pilocytic astrocytoma, which typically occurs in the first decade of life.

Transorbital neuroendoscopic surgery (TONES) as a novel minimally invasive skullbase surgical corridor was described in 2007 (Moe, 2011). The benefits of improved visualization and minimally invasive, endoscopy-assisted surgery have made this corridor attractive for selective conditions affecting the anterior and middle skullbase region.

This report describes a case of histologically confirmed pilocytic astrocytoma in a neonate which presented to our unit as a giant orbital tumor with intracranial extension and also the unique multi-disciplinary team approach and minimally invasive surgical management of the neonate.

## Case description

A new-born boy was referred to our service with the presentation of marked proptosis of the left eye. Magnetic resonance imaging (MRI) revealed an extensive left orbital mass with intracranial extension. A combined, multidisciplinary team approach, including ophthalmology, pediatric neurosurgery, and interventional radiology contributed to optimizing the clinical and surgical outcome in this patient.

### History

The patient was born to a 23-year-old mother with an obstetric history of two previous live births and otherwise no medical history of relevance. The prenatal ultrasound performed at another centre did not apparently reveal any gross abnormalities. A male patient was born at 39-week gestation, delivered by normal vaginal delivery with good Apgar scores and a birth weight of 2.8 kg. On physical examination, a large left eye proptosis was noted at birth, and the patient was then referred to our tertiary level institution for further assessment and management.

### Workup and imaging

**Fig. 1:**
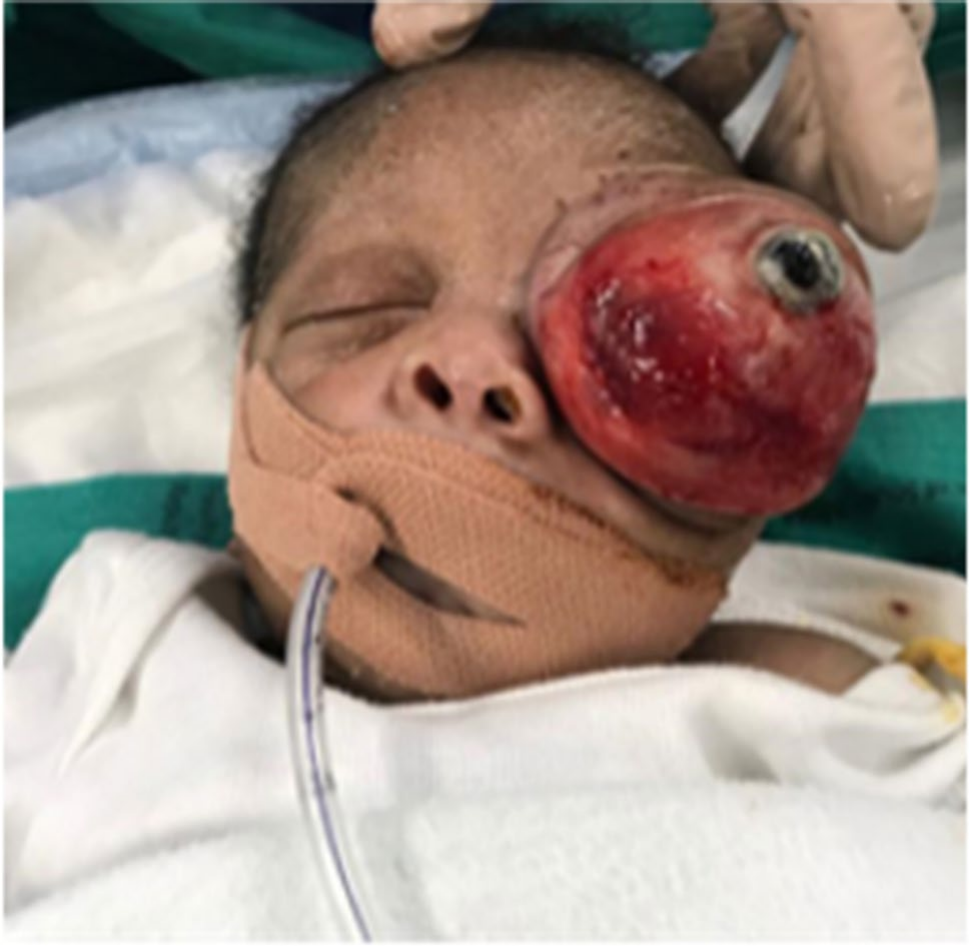
The globe seen anterior to the mass was deformed with complete corneal melt and uveal prolapse

Following transfer to our hospital, an ophthalmologist examined the patient on day 2 of life where an assessment of massive left eye proptosis secondary to a retrobulbar mass was made. The globe seen anterior to the mass was deformed with complete corneal melt and uveal prolapse (Fig. 1). The proptosis was non-pulsatile and separable from the orbital rim and eyelids. The orbit was expanded, but otherwise not infiltrated by the mass. The right eye was assessed to be normal, and the periorbital soft tissues appeared normal. The general examination of the patient demonstrated no other associated abnormalities. The patient had no other dysmorphic features or characteristics suggestive of any neurocutaneous syndromes.

**Fig. 2:**
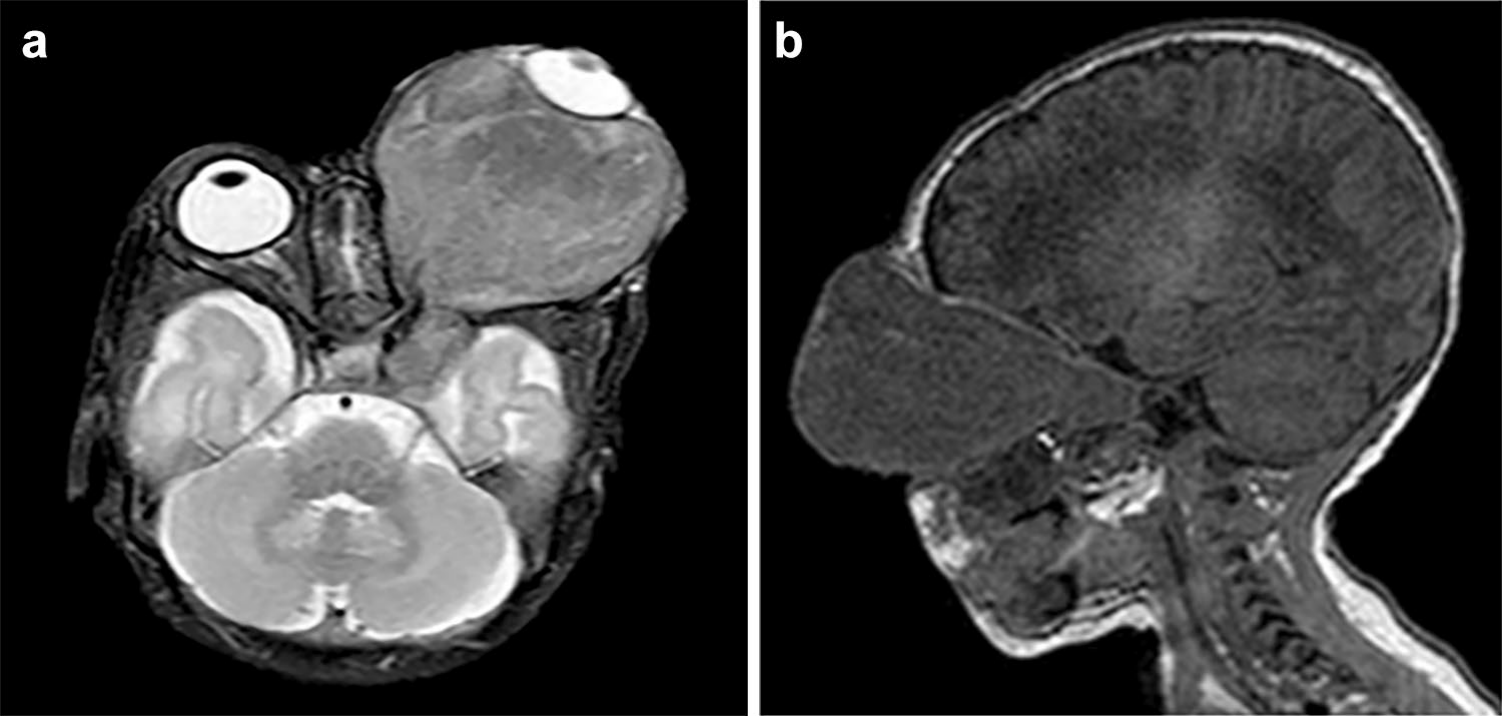
**a** MRI of the head demonstrated a large, well-circumscribed mass in the left orbit. **b** The mass measured 45 mm (anteroposterior) by 47.7 mm (transverse) by 50.9 mm (craniocaudal), with a 25-mm intracranial extension through a widened left optic canal

MRI of the head demonstrated a large, well-circumscribed mass in the left orbit (Fig. 2a) which encased and displaced the left optic nerve and ophthalmic artery medially. The mass was T1W iso-intense and T2W hyperintense to muscle. There was no associated restricted diffusion or blooming to suggest haemorrhage/calcification or fatty components within it. The extraocular muscles could not be identified separately from the mass which anteriorly displaced and flattened the left globe. The mass measured 45 mm (anteroposterior) by 47.7 mm (transverse) by 50.9 mm (craniocaudal), with a 25-mm intracranial extension through a widened left optic canal (Fig. 2b). Posteriorly, the mass abutted the left cavernous sinus without infiltrating the sinus or the internal carotid artery. The rest of the brain was within normal limits. Contrast-enhanced sequences were not performed due to the hospital’s paediatric scan protocol at the time. A preliminary diagnosis of OPG was made.

**Fig. 3:**
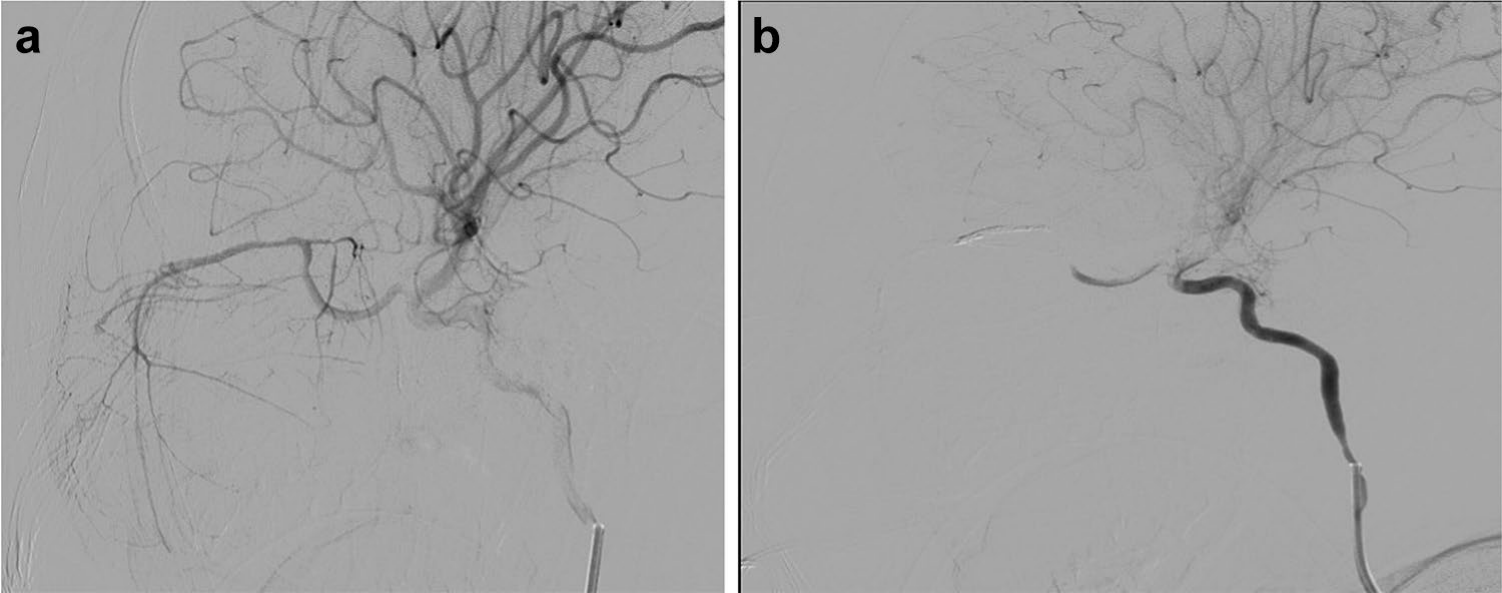
**a** Digital subtraction angiography (DSA) was performed pre-operatively, which demonstrated that the tumor was supplied mainly through the intra-orbital and intra-ocular branches of the ipsilateral ophthalmic artery. **b** This was confirmed post-embolization as there was no supply or tumor blush demonstrated in the tumor area

A presurgical computerized tomography (CT) scan was included to better delineate the bony anatomy and canals, which also confirmed the MRI findings. Digital subtraction angiography (DSA) was performed pre-operatively, which demonstrated that the tumor was supplied mainly through the intra-orbital and intra-ocular branches of the ipsilateral ophthalmic artery (Fig. 3a).

## Surgical management

The initial histological report from an incisional biopsy was inconclusive. A pre-resection embolization of the lesion was then performed co-axially through a microcatheter in the left ophthalmic artery. Onyx material was used to embolize and completely obliterate the left ophthalmic artery and its branches. This was confirmed post-embolization as there was no supply or tumor blush demonstrated in the tumor area (Fig. 3b).

**Fig. 4:**
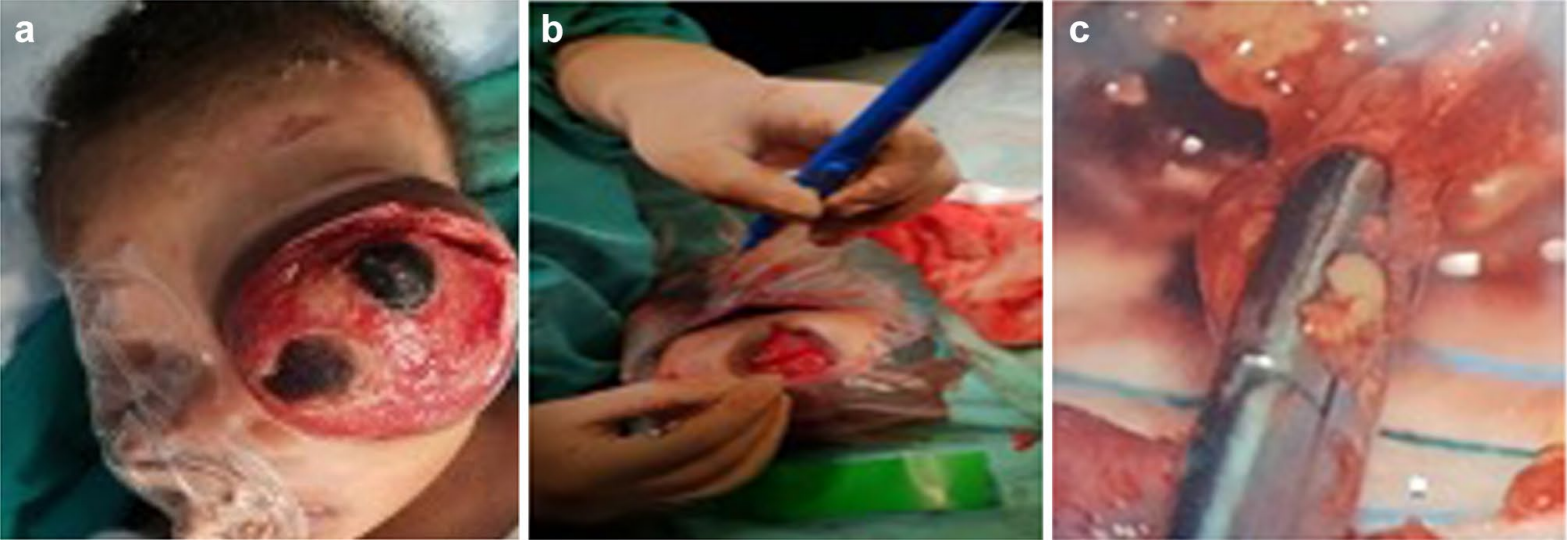
**a**, **b** An orbital lid-sparing exenteration of the tumor was initially performed by the ophthalmologist. **c** The tumor was very well visualised and carefully dissected off the nerve sheath laterally, using a micro-dissector, micro-forceps and suction, under endoscopic guidance

Surgical resection was performed by a multidisciplinary team, including ophthalmology and pediatric neurosurgery. An orbital lid-sparing exenteration of the tumor was initially performed by the ophthalmologist (Fig. 4a, b). There was minimal intra-orbital bleeding. The orbital cavity/volume was noted to be expanded, but the orbital wall bones were not invaded and appeared grossly intact.

**Fig. 5:**
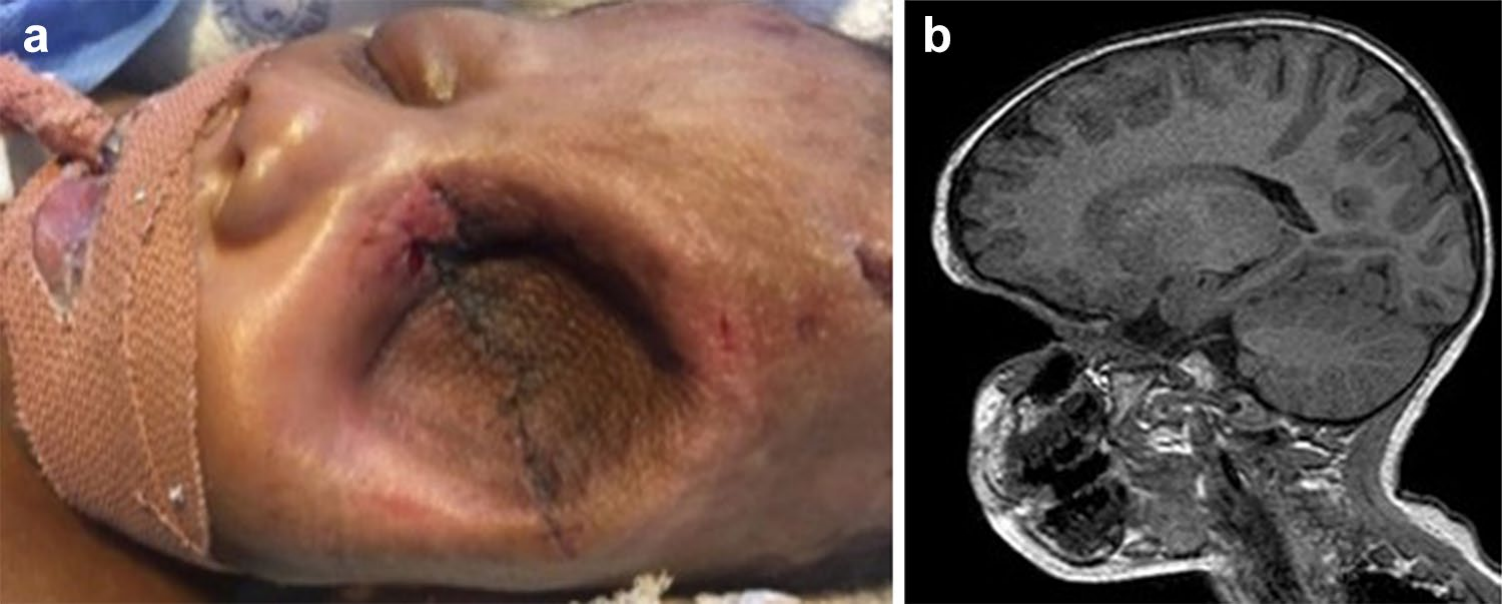
**a** The eyelids were then sutured closed by the ophthalmologist and a small dressing placed over the incision site. **b** The residual mass was demonstrated, comprising of a small intra-canalicular (8 × 5 mm) and intracranial (13 × 8 mm) component, markedly decreased compared to the initial MRI

Following on the resection of the large intra-orbital component of the tumor, the pediatric neurosurgery team then dissected and removed tumor that was exposed through the expanded left optic canal. The intracranial cavity exposure and tumor dissection were performed initially using the 30-degree angled lens on the endoscope (Aesculap, MINOP. This was introduced to assist with visualization of the tumor which extended medially and posteriorly into the intracranial cavity as well as inferiorly into the middle temporal fossa on the left. The tumor was very well visualised and carefully dissected off the nerve sheath laterally, using a micro-dissector, micro-forceps and suction, under endoscopic guidance (Fig. 4c). Minimal bleeding was encountered and was controlled with bipolar on very low settings and surgical haemostat where necessary. The tumor was white, firm, and fibrous in consistency. The intracranial cavity and tumor extension was measured to about 2.5 cm. Circumferentially, the sheath was dissected off, and the dural optic nerve sheath was preserved and remained intact. Surgicel was again placed into the cavity for haemostasis, which was completely dry after resection. The 0-degree endoscope with and without sheath was used to visualize the inferior margin clearly. A small residual segment of tumor, about 8 × 5 mm in size, was intentionally left behind as it medially abutted the cavernous sinus. The eyelids were then sutured closed by the ophthalmologist and a small dressing placed over the incision site (Fig. 5a).

The patient was extubated immediately post-operatively and was admitted to the neonatal intensive care unit for post-operative care. The child recovered very well and was subsequently discharged from the hospital 7 days after surgery.

## Histological features

**Fig. 6:**
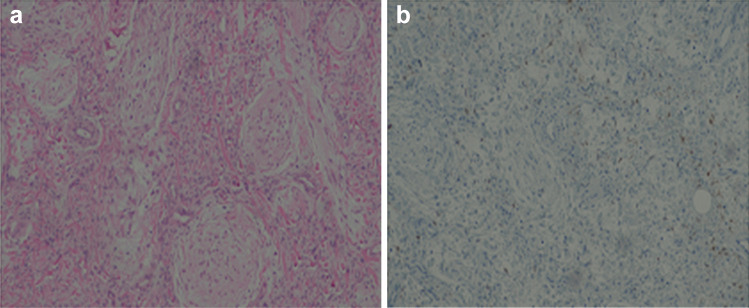
**a** The section was stained with routine haematoxylin and eosin with variable cytology and cellularity, but usually showing overall low-grade elongated hair-like spindle cells often resembling pilocytic astrocytoma. **b** Immunohistochemical stains showed focal-positive immunoreactivity for glial fibrillary acid protein (GFAP) with glial cells

The section was stained with routine haematoxylin and eosin with variable cytology and cellularity, but usually showing overall low-grade elongated hair-like spindle cells often resembling pilocytic astrocytoma (Fig. 6a).

There were Rosenthal fibres (fusiform, cigar-shaped eosinophilic structures within astrocyte cytoplasmic processes) present. These are nonspecific degenerative changes. In addition, there were foci of intense mucinous degeneration with tumor cells in pools of mucin.

Immunohistochemical stains showed focal-positive immunoreactivity for glial fibrillary acid protein (GFAP) with glial cells (Fig. 6b).

The image is a whole slide scan showing cross-sections through the optic nerve that has been expanded by the tumor [3].

Optic nerve glioma may exhibit unusual microscopic features such as extensive arachnoid proliferation, chordoid and chondroid metaplasia, thus mimicking chondrosarcoma or chordoid meningioma. Immunohistochemical staining with GFAP is crucial to exclude these other entities [4].

## Follow-up

The child remained well throughout the hospital stay and was discharged after a week. One month postoperatively, the patient was seen in the neurosurgery outpatient department and was doing really well with complete healing of the incision site with no signs of orbital tumour regrowth or infection.

The patient was lost to follow-up for a period, and then re-presented to the neurosurgery outpatient department 8 months post-surgery, still clinically well with no features of recurrence. A repeat MRI was done at this stage, which demonstrated no typical orbital structures in keeping with left ocular exenteration. The residual mass was demonstrated, comprising of a small intra-canalicular (8 × 5 mm) and intracranial (13 × 8 mm) component, markedly decreased compared to the initial MRI (Fig. 5b). This mass was noted to be heterogeneously contrast-enhancing. The intracranial optic nerve and left optic chiasm were atrophied, and the rest of the brain was within normal limits.

## Discussion

OPG can broadly be categorized into three subtypes, namely childhood syndromic, childhood sporadic and the adult form. Childhood syndromic is associated with neurofibromatosis in about 50% of glioma patients, and their association has been well documented [5]. The occurrence of OPG in non-neurofibromatosis patients tends to produce more severe symptomatology, larger tumor size and distortion of the optic nerve configuration. OPGs rarely occur in the form of malignant glioblastoma of adulthood [6]. The features of pilocytic astrocytoma differ considerably between patients with and without NF1. Our patient presented with an OPG having intracranial extension and no other clinical manifestations suggestive of NF1. He did not meet the diagnostic criteria for NF1 at birth and at age 1 year. It is however important to note that the diagnostic criteria for NF1 are less sensitive in children under the age of 8 than in adults as some of the manifestations develop later in life. Other features also common in non-neurofibromatosis patients are cystic tumour components and hydrocephalus [5]. Neither of these features were demonstrated in our described case.

On imaging findings, the OPG has two primary forms. The more common form causes a fusiform enlargement of the optic pathway, producing an enlarged optic nerve with effacement of the surrounding subarachnoid space. The less common imaging presentation is that of eccentric expansion [5].

Most patients with OPG present between the ages of 2 and 6 years with unilateral loss of vision and axial proptosis, with a mean age of diagnosis at 4–5 years of life. A high index of suspicion is imperative, especially with abnormalities such as optic disc oedema, pallor, atrophy and abnormal pupillary light reflex on clinical examination. Children without neurofibromatosis are likely to present with abnormally increased intracranial pressure, nystagmus and strabismus [1, 7].

The transorbital endoscopic approach provides minimally invasive access to several anatomical regions in the anterior and even middle skullbase regions. This approach may be used as a standalone procedure or in combination with other approaches [8–13]. A unique feature of our case is the expanded optic canal which provided an ideal corridor for endoscopic surgical access to the intracranial component of the tumor, without having to perform any additional bony removal. Our case, to the best of our knowledge, describes the youngest reported case in the literature in which the transorbital surgical approach for intracranial lesion resection was successfully used. The benefit to this child of being spared an additional craniotomy and for limiting blood loss in resecting the intracranial component of this tumor is clear. This serves to underscore the value of minimally invasive surgical techniques for resecting appropriately selected lesions in very young children. There are very limited descriptions of this technique being used in children, although the transorbital approach has been used to access and treat several conditions in adults, including resection of a variety of anterior and middle cranial fossa skullbase tumors, repair of CSF leaks and encephaloceles as well as a feasibility study for hippocampectomy [14–20]. It therefore represents an exciting minimally invasive surgical corridor which will undoubtedly continue to provide pediatric and adult neurosurgeons with an attractive option to access certain intracranial regions and pathologies.

Given the benign profile of this tumor and the location, no adjuvant therapy was given to this child.

## Conclusion

A high index of suspicion and low threshold for further investigation should always be utilised in a child who presents with proptosis, especially as in this case, at birth. The appropriate management of orbital tumors especially in young children requires a multidisciplinary team approach, including obstetricians, paediatricians, ophthalmologists, neurosurgeons, radiologists and pathologists.

Advances in minimally invasive surgical approaches, such as the transorbital endoscopic approach in this case, will continue to enhance the ability of the surgeon to safely access complex lesions through less invasive approaches.

## Data Availability

Not applicable.
